# An interpretable machine learning model for predicting 1-year major adverse cardiovascular events in patients with type 2 diabetes and hypertension

**DOI:** 10.3389/fmed.2026.1871693

**Published:** 2026-07-24

**Authors:** Juan Lv, Xirui Wang, Zhengyi Zhang

**Affiliations:** The Second Hospital and Clinical Medical School, Lanzhou University, Lanzhou, China

**Keywords:** cystatin c, hypertension, machine learning, major adverse cardiovascular events, SHAP interpretability, type 2 diabetes

## Abstract

**Background:**

Patients with coexisting type 2 diabetes mellitus (T2DM) and hypertension (HTN) face a synergistically elevated risk of major adverse cardiovascular events (MACE). Evidence for prediction models developed specifically in established T2DM-HTN comorbidity population remains limited.

**Objective:**

To methodologically explore and preliminarily evaluate an interpretable machine learning framework for 1-year MACE prediction in hospitalized patients with coexisting T2DM and HTN using routine clinical data.

**Methods:**

This retrospective study included 1,054 hospitalized patients with T2DM and HTN, of whom 249 (23.6%) experienced MACE during 1-year follow-up. The dataset was randomly divided into training (60%), validation (20%), and independent test (20%) cohorts using stratified sampling. LASSO regression was applied for feature selection from 69 clinical variables. Four algorithms, including logistic regression, random forest, support vector machine, and XGBoost, were developed and compared. Model performance was assessed using discrimination, calibration, and clinical utility metrics. SHapley Additive exPlanations (SHAP) were used to interpret the final model.

**Results:**

LASSO identified six stable predictors: HbA1c, age, hypertension duration, cystatin C (CysC), T2DM duration, and carotid intima-media thickness (CIMT). Sex was additionally incorporated based on clinical relevance. Multivariable logistic regression showed that HbA1c, age, hypertension duration, T2DM duration, CysC, and CIMT were associated with 1-year MACE risk, whereas sex was not statistically significant. Logistic regression showed the best relative balance between discrimination, calibration, and simplicity on the validation set, although learning curves indicated limited incremental improvement with increasing training sample size. After isotonic regression recalibration, the final logistic regression model achieved an ROC-AUC of 0.828, a PR-AUC of 0.656, and a Brier score of 0.116 on the independent test set. Decision curve analysis indicated potential clinical net benefit. SHAP linked model predictions to glycemic burden, aging, cumulative disease exposure, renal-related risk, and subclinical atherosclerosis.

**Conclusion:**

An interpretable logistic regression model based on seven routine clinical variables showed relatively good internal performance for predicting 1-year composite MACE risk in hospitalized patients with coexisting T2DM and HTN. CysC provided additional prognostic information beyond its conventional role as a renal filtration marker, although this association should be interpreted as prognostic rather than causal. External validation is required before the model can be considered for clinical decision support.

## 1 Introduction

Type 2 diabetes mellitus (T2DM) and hypertension (HTN) are the most common cardiometabolic diseases worldwide, with over 60% of individuals with T2DM also having HTN (1). In a comparative longitudinal analysis of the CHARLS and HRS cohorts, hypertension and diabetes showed a significant additive interaction for cardiovascular events, with approximately 28–31% of the excess risk attributable to their interaction (2). The coexistence of these two conditions confers a substantially elevated cardiovascular risk and may exert an excess effect beyond that expected from either condition alone, driven by synergistic pathophysiological mechanisms including insulin resistance, endothelial dysfunction, chronic low-grade inflammation, and aberrant activation of the renin-angiotensin-aldosterone system (3–6). Sustained hyperglycemia promotes the accumulation of advanced glycation end-products and amplifies oxidative stress, leading to endothelial injury and adverse vascular remodeling, while elevated blood pressure imposes direct hemodynamic stress on the arterial wall, further accelerating atherosclerosis (7–9). In China, the prevalence of T2DM and HTN is particularly pronounced in the economically underdeveloped western regions (10), underscoring the urgent need for precise, regionally adapted risk stratification tools to enable early identification of high-risk individuals and efficient allocation of preventive resources.

Existing cardiovascular risk prediction models, including the Framingham Risk Score (11), the SCORE project (12), QRISK (13), and the China-PAR model (14), are predominantly derived from general populations and are unable to fully capture the complex interactions among metabolic, hemodynamic, and renal variables in patients with comorbidities (15, 16). In patients with coexisting T2DM and HTN, interrelated risk factors often exhibit significant collinearity and nonlinear associations with clinical outcomes, limiting the discriminative performance of traditional regression models (17, 18).

Machine learning (ML) algorithms—such as Random Forest (RF) and eXtreme Gradient Boosting (XGBoost)—offer powerful alternatives by automatically detecting nonlinear relationships and high-order interactions from high-dimensional clinical data without a priori assumptions (19–21). However, the “black-box” nature of many high-performance ML models has hindered their clinical translation, as clinicians are rightly hesitant to make treatment decisions based on uninterpretable risk scores (22, 23). The introduction of SHapley Additive exPlanations (SHAP) has begun to address this barrier by quantifying the contribution of each feature to an individual prediction, enabling both global and local model interpretability (24, 25).

Despite these methodological advances, a fundamental question remains: among the routinely collected clinical variables available in hospitalized patients with coexisting T2DM and HTN, which variables contain reproducible prognostic information for short-term composite MACE risk? This study did not prespecify a specific biomarker hypothesis, but instead adopted a data-driven exploratory approach by applying LASSO regression to a broad pool of routine clinical variables, including demographics, vital signs, hematologic indices, metabolic profile, hepatic and renal function, electrolytes, medication information, and CIMT. The selected variables were then used to develop and compare several candidate machine learning models, with SHAP applied to improve interpretability of the final model. Given the retrospective single-center design and the observed limitations in learning dynamics, this study should be regarded as a methodological exploration and hypothesis-generating analysis rather than as the development of a model ready for clinical deployment.

Accordingly, this study had three objectives: (1) to identify candidate predictors of 1-year composite MACE using LASSO; (2) to develop and compare several machine learning models under a training–validation–test framework; and (3) to use SHAP to interpret model predictions and generate clinically plausible hypotheses for future validation in patients with coexisting T2DM and HTN.

## 2 Materials and methods

### 2.1 Study participants

**FIGURE 1:**
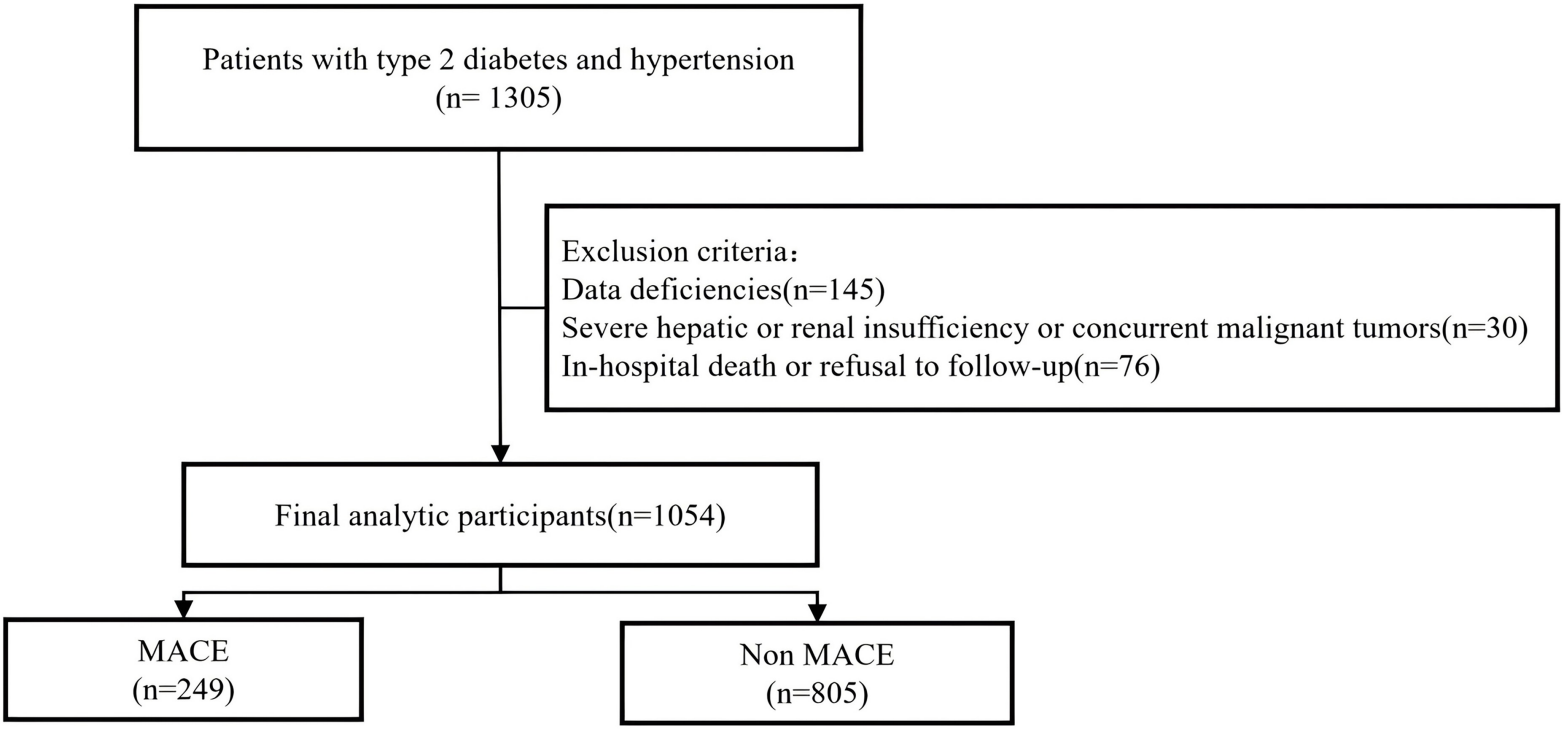
The flowchart of study participants.

This retrospective study included patients with T2DM and HTN in the Endocrine Department at the Second Hospital of Lanzhou University between October 2023 and October 2024. Inclusion criteria: (1) age ≥ 18years; (2) meeting the diagnostic criteria for T2DM and HTN. Exclusion criteria included: incomplete clinical data; severe hepatic or renal insufficiency; coexisting malignant tumors; and in-hospital death or refusal to participate in follow-up. This retrospective study was approved by the Ethics Committee of the Second Hospital of Lanzhou University (Figure 1).

### 2.2 Study follow-up

Patients were followed up by telephone interviews and outpatient clinic visits to assess clinical outcomes within 1 year after hospital discharge. The endpoint was major adverse cardiovascular events (MACE), defined as a composite of all-cause death, myocardial infarction, stroke, heart failure, and related rehospitalization. All MACE events were independently adjudicated by two physicians through a comprehensive review of medical records, discharge summaries, and follow-up data, with a Cohen’s Kappa coefficient of 0.85 (95% CI: 0.81–0.89). Any discrepancies between adjudicators were resolved by consensus discussion to reach a final decision. Patients were divided into MACE (*n* = 249) and non-MACE (*n* = 805) groups based on follow-up outcomes (26, 27).

### 2.3 Data collection

Data on baseline characteristics were collected at the time of hospital admission and included the following categories:

(1) Demographics: age, sex, ethnicity, and marital status.

(2) Lifestyle and physical examination: smoking and drinking history, body temperature, heart rate, height, weight, body mass index (BMI), systolic blood pressure (SBP), and diastolic blood pressure (DBP).

(3) Medical history: duration of type 2 diabetes mellitus (T2DM) and hypertension (HTN).

(4) Laboratory parameters, including: (a) Complete blood count: white blood cell (WBC), neutrophil, lymphocyte, monocyte, eosinophil, basophil, red blood cell (RBC), hemoglobin (HGB), hematocrit (HCT), mean corpuscular volume (MCV), and platelet (PLT). (b) Inflammatory markers: C-reactive protein (CRP), systemic inflammatory response index (SIRI), and systemic immune inflammatory index (SII). (c) Glycemic and metabolic profile: hemoglobin A1c (HbA1c), fasting serum glucose (GLU), and triglyceride-glucose (TyG) index. (d) Lipid profile: total cholesterol (TC), triglyceride (TG), high-density lipoprotein cholesterol (HDL-c), low-density lipoprotein cholesterol (LDL-c), and atherogenic index of plasma (AIP). (e) Liver function: alanine aminotransferase (ALT), aspartate aminotransferase (AST), gamma-glutamyl transferase (GGT), alkaline phosphatase (ALP), serum total protein (STP), albumin (ALB), total bilirubin (Tbil), direct bilirubin (Dbil), and indirect bilirubin (Ibil). (f) Renal function: blood urea nitrogen (BUN), creatinine (Cr), uric acid (UA), cystatin C (CysC), and estimated glomerular filtration rate (eGFR). Chronic kidney disease (CKD) status was also recorded. (g) Myocardial and other markers: creatine kinase (CK), creatine kinase isoenzyme (CK-MB), lactate dehydrogenase (LDH), and homocysteine (Hcy). (h) Electrolytes: potassium (K^+^), sodium (Na^+^), chloride (Cl^–^), calcium (Ca^2+^), phosphorus (P), and magnesium (Mg^2+^).

(5) Medication history: use of antihyperglycemic agents, antihypertensive agents, insulin, antiplatelet drugs, and lipid-lowering drugs at admission.

(6) Auxiliary examination: carotid intima-media thickness (CIMT).

In total, 69 original clinical variables were collected. After categorical variables were dummy-encoded, these variables were represented by 79 predictor features for modeling. Together with the binary MACE outcome variable, the analytical dataset therefore comprised 80 columns in total. The MACE outcome variable was retained solely as the response variable and was not included among the predictor features.

### 2.4 Statistical analyses

All analyses were conducted using R software (version 4.5.3) and Python (version 3.14.3). Baseline characteristics were compared between the MACE and non-MACE groups. Continuous variables are presented as mean ± standard deviation or median (interquartile range) and compared using the Student’s *t*-test or Mann-Whitney U test, as appropriate. Categorical variables are presented as frequencies (percentages) and compared using the chi-squared test. Statistical significance was set at a two-tailed *P* < 0.05.

### 2.5 Data preprocessing

The original dataset was randomly divided into training (60%), validation (20%), and test (20%) cohorts using a stratified sampling strategy to preserve the proportion of the primary outcome (MACE) across all subsets. The training set was used for feature selection, model development and learning curves, the validation set was used for model comparison, and the test set was reserved exclusively for final performance evaluation.

Continuous variables were standardized using z-score normalization based solely on the training dataset to prevent information leakage. The same scaling parameters were subsequently applied to the validation and test sets. Categorical variables were dummy-encoded with the first category as the reference level.

Class imbalance was mitigated within the training data only. SMOTE was applied before fitting logistic regression, random forest, and SVM models, whereas XGBoost used the native scale_pos_weight parameter calculated from the non-MACE/MACE ratio in the training set. The validation and test sets were not resampled.

### 2.6 Feature selection with LASSO

To identify the most robust predictors of MACE from the pool of collected variables, the Least Absolute Shrinkage and Selection Operator (LASSO) algorithm was applied on the training set using 10-fold cross-validation. The penalty parameter lambda (λ) was optimized using the 1-SE (one standard error) criterion, which was pre-specified in our analytical plan to enhance generalizability and reduce overfitting by choosing the largest λ within one standard error of the minimum binomial deviance (λ.1se). The final set of predictors was those whose coefficients remained non-zero at this optimal λ.1se, effectively performing variable selection.

### 2.7 Interpretive model development

Variables selected by LASSO were subsequently entered into a multivariable logistic regression model to quantify their independent association with MACE via odds ratios (OR) and 95% confidence intervals (CI).

### 2.8 Predictive model development and performance evaluation

Four machine learning algorithms were developed and compared in this study, including Logistic Regression (LR), Random Forest (RF), Support Vector Machine (SVM), and eXtreme Gradient Boosting (XGBoost).

All models were trained using the training cohort, and hyperparameters were optimized exclusively within the training set using grid search combined with 5-fold cross-validation. The validation set was not involved in any stage of model training or hyperparameter tuning, ensuring strict independence between model development and evaluation.

For Logistic Regression, L2 regularization was applied to control model complexity. For Random Forest, key hyperparameters including the number of trees, maximum tree depth, and minimum samples per leaf were optimized. For SVM, a radial basis function (RBF) kernel was used, with tuning of the cost and gamma parameters. For XGBoost, learning rate, maximum tree depth, subsampling ratio, and number of estimators were optimized using cross-validation.

To evaluate the training stability and generalization capability of each machine learning algorithm prior to formal performance comparison, learning curves were constructed on the training set across incremental training sample sizes (ranging from 10 to 100% of the training data in 10% increments). For each subsample, model performance (measured by accuracy and log-loss) was calculated on both the training and validation subsets. This approach allowed us to assess whether the models exhibited overfitting (indicated by a persistent gap between training and validation performance) and to determine whether more complex algorithms would benefit from additional data or, conversely, whether simpler models already achieved stable and generalizable performance.

Model performance was evaluated on the independent validation set using multiple metrics, including discrimination (AUC), calibration (Brier score), and classification performance indicators. Bootstrap resampling with 2,000 iterations was employed to construct 95% confidence intervals (CIs) for all performance metrics.

The final model’s performance on the independent test set was evaluated with a comprehensive suite of metrics, including AUC, smoothed Precision-Recall (PR) curves, Brier scores, calibration curves, and Decision Curve Analysis (DCA).

### 2.9 Model interpretation using SHAP

SHAP (SHapley Additive exPlanations) were additionally applied to provide a unified feature attribution framework consistent with machine learning interpretability standards. This method quantifies the impact of each feature on individual predictions, providing both global and local interpretability. The analysis generated SHAP summary plots to rank feature importance and show the direction of their effects, and integrated visualizations to provide a comprehensive view of feature-attribution patterns.

## 3 Results

### 3.1 Baseline characteristics

A total of 1,054 eligible patients (mean age 62 ± 11 years, 62.0% male) were enrolled and completed the 1-year follow-up, among whom 249 (23.6%) experienced MACE. Compared to the non-MACE group, patients in the MACE group were significantly older, had a longer median duration of diabetes and hypertension, and higher levels of SBP, HbA1c, CysC, and Scr (all *P* < 0.05). Conversely, the MACE group had significantly lower DBP, BMI, ALB, and eGFR (all *P* < 0.05). Furthermore, the utilization rates of insulin, antiplatelet drugs, and lipid-lowering agents were significantly higher in the MACE group (all P < 0.05). The detailed baseline characteristics of the study population are presented in Table 1. All *p*-values are solely for descriptive analysis and should not be used to infer independent predictive relationships.

**TABLE 1:** Baseline data comparison between groups.

Variables	Total (*n* = 1,054)	Non-MACE (*n* = 805)	MACE (*n* = 249)	*P*-value
Demographics				
Age, years	62 ± 11	59 ± 11	69 ± 9	< 0.001
Male, n (%)	653 (62.0)	508 (63.1)	145 (58.2)	0.166
ıHan nationality, n (%)	913 (86.6)	697 (86.6)	216 (86.7)	0.780
Married, n (%)	1038 (98.5)	794 (98.6)	244 (98.0)	0.552
Lifestyle and physical examination				
Smoking, n (%)	158 (15.0)	125 (15.5)	33 (13.3)	0.379
Drinking, n (%)	63 (6.0)	57 (7.1)	6 (2.4)	0.007
Body temperature, °C	36.29 ± 0.20	36.29 ± 0.20	36.28 ± 0.19	0.374
Heart rate, times/min	84 ± 13	85 ± 13	81 ± 13	< 0.001
Height, cm	167 ± 8	167 ± 8	166 ± 8	0.015
Weight, kg	70 ± 12	71 ± 12	68 ± 11	< 0.001
BMI, kg/m^2^	24.8 (22.9, 26.9)	24.8 (22.9, 27.1)	24.2 (22.5, 26.3)	0.023
SBP, mmHg	134 (129, 139)	134 (129, 138)	135 (131, 140)	0.014
DBP, mmHg	82 (77, 86)	83 (78, 87)	79 (75, 82)	< 0.001
Medical history				
Type 2 diabetes duration, years	10 (4, 17)	8 (3, 15)	16 (8, 20)	< 0.001
Hypertension duration, years	8 (3, 12)	7 (3, 10)	10 (7, 20)	< 0.001
Complete blood count				
WBC, 10^9^/L	6.31 (5.20, 7.40)	6.27 (5.17, 7.34)	6.60 (5.50, 7.69)	0.026
Neutrophil, 10^9^/L	4.03 (3.25, 4.98)	3.92 (3.19, 4.89)	4.40 (3.52, 5.24)	< 0.001
Lymphocyte, 10^9^/L	1.61 (1.29, 2.00)	1.65 (1.31, 2.05)	1.52 (1.18, 1.86)	< 0.001
Monocyte, 10^9^/L	0.38 (0.30, 0.47)	0.37 (0.29, 0.46)	0.39 (0.31, 0.49)	0.047
Eosinophils, 10^9^/L	0.10 (0.06, 0.16)	0.10 (0.06, 0.16)	0.10 (0.06, 0.15)	0.307
Basophils, 10^9^/L	0.03 (0.02, 0.04)	0.03 (0.02, 0.04)	0.03 (0.02, 0.04)	0.110
RBC, 10^12^/L	4.97 ± 0.63	5.01 ± 0.63	4.81 ± 0.62	< 0.001
HGB, g/L	153 (140, 165)	155 (142, 167)	147 (135, 159)	< 0.001
HCT, %	0.46 (0.42, 0.49)	0.46 (0.43, 0.50)	0.44 (0.41, 0.48)	< 0.001
MCV, L/L	92.0 (89.2, 94.8)	92.1 (89.3, 94.9)	91.4 (88.5, 94.5)	0.124
PLT, 10^9^/L	197 (164, 244)	196 (165, 242)	201 (158, 246)	0.787
Inflammatory markers				
CRP, mg/L	2.4 (1.2, 4.1)	2.2 (1.1, 3.7)	2.9 (1.4, 5.5)	< 0.001
SIRI	0.95 (0.63, 1.38)	0.91 (0.60, 1.31)	1.09 (0.75, 1.63)	< 0.001
SII	497 (353, 691)	477 (337, 660)	569 (422, 781)	< 0.001
Glycemic and metabolic profile				
HbA1c, %	8.70 (7.90, 9.60)	8.40 (7.80, 9.30)	9.80 (9.00, 10.70)	< 0.001
GLU, mmol/L	9.4 (7.1, 12.8)	8.9 (7.0, 11.6)	11.3 (8.0, 14.9)	< 0.001
TyG	9.48 ± 0.83	9.44 ± 0.82	9.58 ± 0.84	0.024
Lipid profile				
TC, mmol/L	4.47 ± 1.29	4.50 ± 1.28	4.39 ± 1.30	0.241
TG, mmol/L	1.58 (1.10, 2.38)	1.59 (1.10, 2.37)	1.53 (1.12, 2.41)	0.916
HDL-c, mmol/L	1.15 ± 0.27	1.15 ± 0.27	1.15 ± 0.27	0.907
LDL-c, mmol/L	2.83 ± 0.91	2.85 ± 0.89	2.78 ± 0.98	0.328
AIP	0.41 ± 0.70	0.41 ± 0.72	0.38 ± 0.63	0.501
Liver function				
ALT, U/L	22 (16, 32)	23 (16, 34)	18 (13, 26)	< 0.001
AST, U/L	23 (19, 30)	24 (19, 30)	22 (18, 28)	< 0.001
GGT, U/L	25 (17, 39)	26 (17, 41)	21 (16, 36)	0.004
ALP, U/L	92 ± 28	91 ± 28	98 ± 29	< 0.001
STP, g/L	73.7 ± 6.1	73.8 ± 5.9	73.0 ± 6.8	0.088
ALB, g/L	43.2 ± 4.3	43.9 ± 4.0	41.1 ± 4.7	< 0.001
Tbil, μmol/L	14.4 (11.4, 18.4)	14.7 (11.5, 18.7)	13.6 (10.5, 17.5)	0.003
Dbil, μmol/L	2.80 (2.00, 3.70)	2.90 (2.00, 3.80)	2.70 (1.90, 3.70)	0.033
Ibil, μmol/L	12.6 ± 5.3	12.9 ± 5.5	11.7 ± 4.7	0.001
Renal function				
BUN, mmol/L	6.58 ± 2.44	6.37 ± 2.34	7.28 ± 2.62	< 0.001
Cr, μmol/L	66 (54, 79)	65 (53, 77)	71 (58, 90)	< 0.001
UA, μmol/L	328 (273, 392)	330 (274, 394)	318 (266, 376)	0.053
CysC, mg/L	0.98 (0.88, 1.15)	0.95 (0.86, 1.09)	1.12 (0.99, 1.31)	< 0.001
eGFR, mL/min/1.73 m^2^	89.16 ± 26.40	93.52 ± 25.72	75.06 ± 23.53	< 0.001
CKD stage, n (%)				< 0.001
G1	556 (52.8)	486 (60.4)	70 (28.1)	
G2	330 (31.3)	219 (27.2)	111 (44.6)	
G3a	101 (9.6)	65 (8.1)	36 (14.5)	
G3b	53 (5.0)	29 (3.6)	24 (9.6)	
G4	14 (1.3)	6 (0.7)	8 (3.2)	
CKD, n (%)	168 (15.9)	100 (12.4)	68 (27.3)	< 0.001
Myocardial and other markers				
CK, U/L	104 ± 122	106 ± 134	96 ± 71	0.129
CK-MB, U/L	14.2 ± 9.4	13.8 ± 7.5	15.5 ± 13.8	0.070
LDH, U/L	213 ± 54	211 ± 55	220 ± 49	0.011
Hcy, μmol/L	17.4 ± 4.0	17.3 ± 4.1	17.8 ± 3.8	0.070
Electrolytes				
K^+^, mmol/L	3.83 ± 0.43	3.81 ± 0.41	3.89 ± 0.48	0.028
Na^+^, mmol/L	136.72 ± 2.75	136.95 ± 2.52	135.96 ± 3.28	< 0.001
Cl^–^, mmol/L	102.5 ± 3.3	102.7 ± 3.1	101.9 ± 3.5	0.002
Ca^2+^, mmol/L	2.35 ± 0.11	2.36 ± 0.10	2.33 ± 0.11	< 0.001
P, mmol/L	1.06 ± 0.18	1.06 ± 0.18	1.06 ± 0.18	0.860
Mg^2+^, mmol/L	0.86 ± 0.09	0.86 ± 0.09	0.87 ± 0.08	0.026
Medication use				
Number of antihyperglycemic agents, n (%)				0.073
0	36 (3.4)	23 (2.9)	13 (5.2)	
1	131 (12.4)	92 (11.4)	39 (15.7)	
2	270 (25.6)	208 (25.8)	62 (24.9)	
≥ 3	617 (58.5)	482 (59.9)	135 (54.2)	
Number of antihypertensive agents, n (%)				< 0.001
0	157 (14.9)	136 (16.9)	21 (8.4)	
1	340 (32.3)	278 (34.5)	62 (24.9)	
2	269 (25.5)	202 (25.1)	67 (26.9)	
≥ 3	288 (27.3)	189 (23.5)	99 (39.8)	
Insulin use, n (%)	723 (68.6)	500 (62.1)	223 (89.6)	< 0.001
Antiplatelet drug use, n (%)	586 (55.6)	404 (50.2)	182 (73.1)	< 0.001
Lipid-lowering drug use, n (%)	856 (81.2)	639 (79.4)	217 (87.1)	0.006
Auxiliary examination				
CIMT, mm	1.05 ± 0.11	1.04 ± 0.11	1.10 ± 0.10	< 0.001

Data are presented as mean ± SD, median (Q1, Q3), or n (%). BMI, body mass index; SBP, systolic blood pressure; DBP, diastolic blood pressure; WBC, white blood cell; RBC, red blood cell; HGB, hemoglobin; HCT, hematocrit; MCV, mean corpuscular volume; PLT, platelet; CRP, C-reactive protein; SIRI, systemic inflammatory response index; SII, systemic immune inflammatory index; HbA1c, hemoglobin A1c; GLU, serum glucose; TyG, triglyceride-glucose index; TC, total cholesterol; TG, triglyceride; HDL-c, high-density lipoprotein cholesterol; LDL-c, low-density lipoprotein cholesterol; AIP, atherogenic index of plasma; ALT, alanine aminotransferase; AST, aspartate aminotransferase; GGT, gamma-glutamyl transferase; ALP, alkaline phosphatase; STP, serum total protein; ALB, albumin; Tbil, total bilirubin; Dbil, direct bilirubin; Ibil, indirect bilirubin; BUN, blood urea nitrogen; Cr, creatinine; UA, uric acid; CysC, cystatin C; eGFR, estimated glomerular filtration rate; CKD, chronic kidney disease; CK, creatine kinase; CK-MB, creatine kinase isoenzyme; LDH, lactate dehydrogenase; Hcy, homocysteine; CIMT, carotid intima-media thickness.

### 3.2 Variable selection via LASSO

**FIGURE 2:**
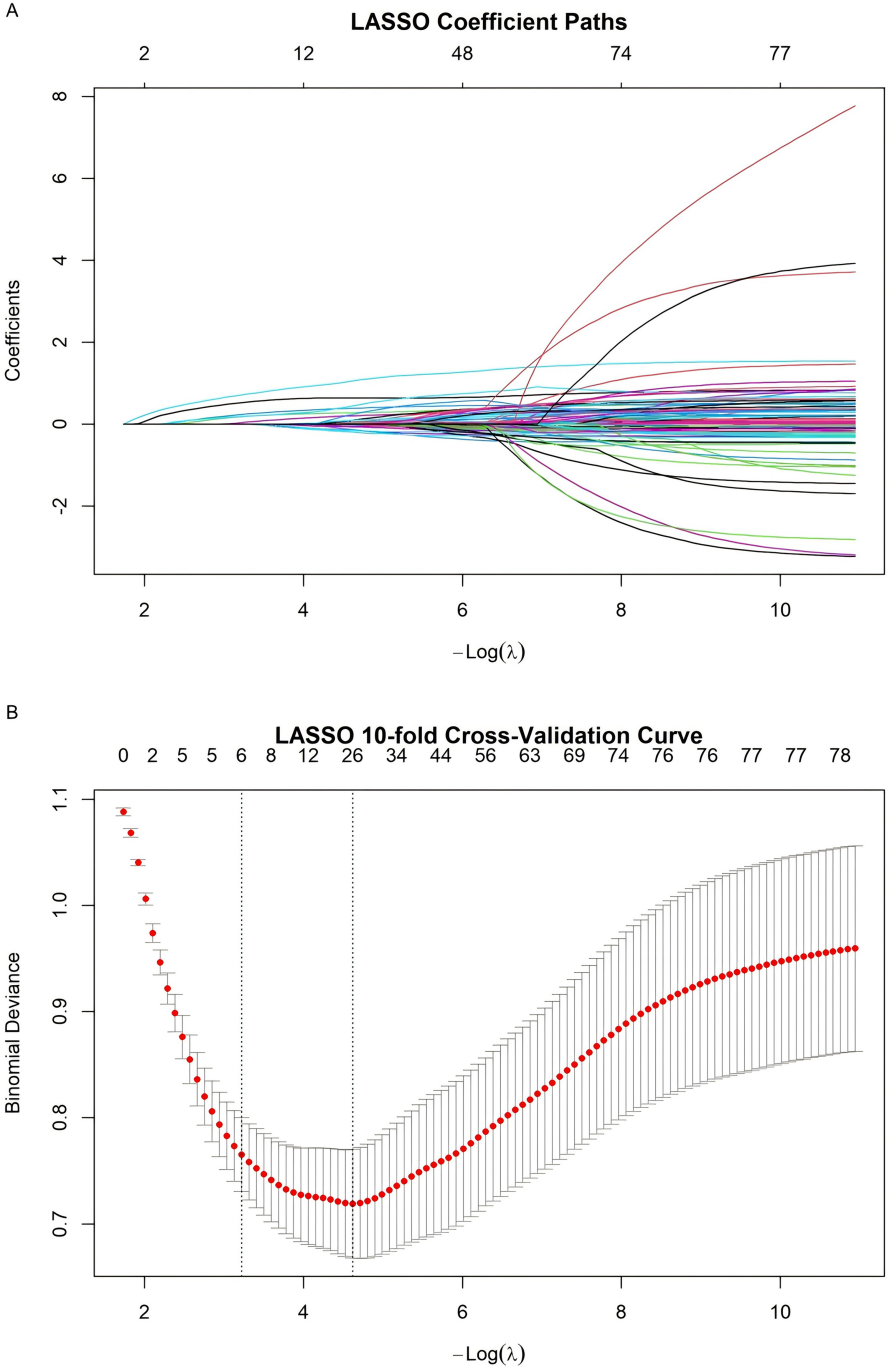
LASSO coefficient paths and cross-validation. **(A)** Complete coefficient paths across -log(λ). As λ increases, the regression coefficients of each variable gradually contract to zero. **(B)** Ten-fold CV misclassification error (red dots) ± 1 SE (gray bars) plotted against log(λ). Left dashed line = λ_1se (largest λ within one SE); right dashed line = λ_min (min CV error).

LASSO regression with 10-fold cross-validation was applied for feature selection (Figure 2). Two penalization parameters were considered: the minimum cross-validated error (λ.min = 0.0099) and the more parsimonious one-standard-error criterion (λ.1se = 0.0399), which was selected for final model development to enhance generalizability and reduce overfitting.

At λ.min, a total of 26 predictors retained non-zero coefficients, representing a broader set of potentially informative variables. These included: HbA1c, age, cysC, hypertension duration, type 2 diabetes duration, CIMT, CKD stage, GGT, heart rate, UA, Hcy, STP, Mg^2 +^, WBC, Tbil, HDL-C, K ^+^, Cl^–^, body temperature, CK-MB, height, Cr, LDL-C, AIP, M, and Ibil.

At λ.1se, six stable predictors remained, including HbA1c, age, hypertension duration, cystatin C, type 2 diabetes duration, and CIMT. These variables were therefore selected as the final predictors for multivariable modeling. Given its established clinical significance, Sex was also included based on expert clinical judgment. Consequently, a final set of seven variables was used for subsequent multivariable analysis and model development.

### 3.3 Multivariable logistic regression analysis

Multivariable logistic regression analysis were associated with 1-year MACE, with CIMT showing borderline significance (Table 2). After standardization, HbA1c emerged as the strongest predictor (OR = 3.06, 95% CI: 2.33–4.03, *P* < 0.001), with each standard deviation increase associated with a threefold elevation in MACE risk. Age was also a strong predictor (OR = 2.26, 95% CI: 1.66–3.07, *P* < 0.001). Longer hypertension duration (OR = 1.63, 95% CI: 1.28–2.09, *P* < 0.001) and type 2 diabetes duration (OR = 1.52, 95% CI: 1.20–1.93, *P* = 0.001) were significantly associated with increased risk, reflecting the cumulative burden of chronic disease. Higher CysC (OR = 1.39, 95% CI: 1.11–1.73, *P* = 0.004) and CIMT (OR = 1.36, 95% CI: 1.00–1.86, *P* = 0.050) were also significant predictors, with CIMT exhibiting borderline significance. Sex was not a statistically significant predictor (OR = 0.89, 95% CI: 0.54–1.47, *P* = 0.644).

**TABLE 2:** Predictors of MACE from binary multivariable logistic regression analysis.

Predictor	OR	95% CI lower	95% CI upper	Estimate (β)	Std. error	*Z*-value	*P*-value
HbA1c (%)	3.06	2.33	4.03	1.1196	0.1397	8.0138	< 0.001
Age (years)	2.26	1.66	3.07	0.8143	0.1571	5.1830	< 0.001
Hypertension duration (years)	1.63	1.28	2.09	0.4909	0.1251	3.9273	< 0.001
Type 2 diabetes duration (years)	1.52	1.20	1.93	0.4217	0.1214	3.4730	0.001
CysC (mg/L)	1.39	1.11	1.73	0.3288	0.1134	2.9009	0.004
CIMT (mm)	1.36	1.00	1.86	0.3095	0.1582	1.9570	0.050
Sex (Female)	0.89	0.54	1.47	-0.1186	0.2569	-0.4617	0.644

β beta coefficient from logistic regression, Std. Error Standard Error.

### 3.4 Learning curve analysis

**FIGURE 3:**
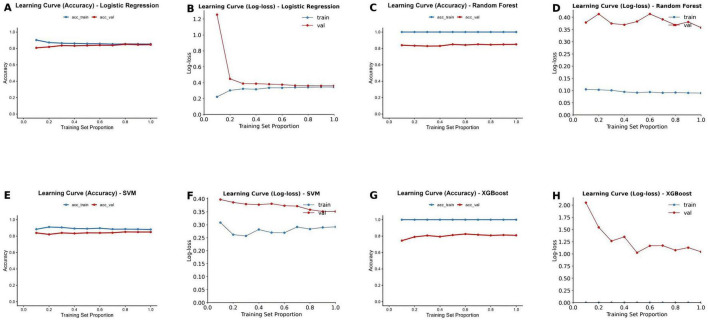
**(A,B)** Logistic regression. **(C,D)** Random forest. **(E,F)** Support vector machine. **(G,H)** XGBoost. **(A,C,E, G)** Accuracy-based learning curves, while **(B,D,F,H)** present log-loss-based learning curves. Blue lines represent performance on the training set, and red lines represent performance on the validation set. The x-axis indicates the proportion of training data used, while the y-axis represents model performance (accuracy or log-loss).

To evaluate model training behavior and potential generalizability, learning curves were constructed for the four candidate algorithms, including logistic regression, random forest, support vector machine, and XGBoost (Figure 3). Accuracy and log-loss were assessed across increasing training sample sizes.

The learning curves revealed important limitations in model learning dynamics. For logistic regression, the training curve remained slightly above the validation curve, which is expected and does not by itself indicate overfitting. However, the validation accuracy increased only modestly as the training sample size increased, suggesting that the model rapidly saturated the predictive signal available from the included variables. Therefore, the relatively flat logistic regression learning curve should be interpreted as limited incremental learning from additional data rather than as definitive evidence of robust generalization.

Random forest and XGBoost showed more pronounced divergence between training and validation performance, consistent with overfitting and limited transportability in this dataset. The support vector machine model showed no meaningful improvement in validation performance with increasing sample size, suggesting that additional training data within the current feature set did not substantially improve its predictive ability.

Overall, none of the four candidate algorithms exhibited completely red-flag-free learning dynamics. Logistic regression was selected as the final model because it showed the best relative balance between discrimination, calibration, and simplicity among the evaluated models, but the learning curve findings indicate that the model’s generalizability should be interpreted cautiously.

### 3.5 Model performance comparison

The predictive performance of the four machine learning models was evaluated on the validation set using multiple metrics, including AUC, accuracy, sensitivity, specificity, PPV, NPV, F1-score, and Brier score (Table 3).

**TABLE 3:** Performance comparison of four models on the validation set.

Model	Performance metric							
	AUC	Brier score	Accuracy	Sensitivity	Specificity	PPV	NPV	F1 score
LR	0.910 (0.855–0.955)	0.089 (0.064–0.116)	0.891 (0.844–0.934)	0.720 (0.588–0.842)	0.944 (0.907–0.976)	0.800 (0.674–0.913)	0.916 (0.871–0.956)	0.758 (0.651–0.848)
RF	0.893 (0.836–0.940)	0.101 (0.075–0.129)	0.867 (0.820–0.915)	0.620 (0.480–0.755)	0.944 (0.906–0.976)	0.775 (0.639–0.900)	0.889 (0.840–0.935)	0.689 (0.565–0.788)
SVM	0.896 (0.845–0.941)	0.103 (0.079–0.129)	0.854 (0.806–0.900)	0.621 (0.489–0.760)	0.926 (0.883–0.964)	0.722 (0.580–0.853)	0.888 (0.839–0.933)	0.665 (0.551–0.773)
XGBoost	0.849 (0.766–0.916)	0.115 (0.086–0.148)	0.858 (0.806–0.900)	0.660 (0.522–0.792)	0.919 (0.874–0.958)	0.715 (0.578–0.844)	0.897 (0.848–0.941)	0.684 (0.565–0.783)

LR, Logistic Regression; RF, Random Forest; SVM, Support Vector Machine. Values are presented as estimate (95% confidence interval).

Among the models, logistic regression demonstrated the most balanced performance, with high discrimination and the best calibration. Random forest and SVM showed comparable but slightly inferior performance. Although XGBoost achieved competitive discrimination, its calibration performance was suboptimal, as reflected by a higher Brier score.

Overall, logistic regression achieved the best balance between discrimination and calibration.

### 3.6 Performance of the final model

Based on the validation-set comparison, logistic regression was selected as the final model because it achieved relatively favorable balance between discrimination, calibration, and model simplicity among the evaluated algorithms. However, as indicated by the learning curve analysis, this selection should be interpreted cautiously because none of the candidate models demonstrated fully optimal learning dynamics.

**FIGURE 4:**
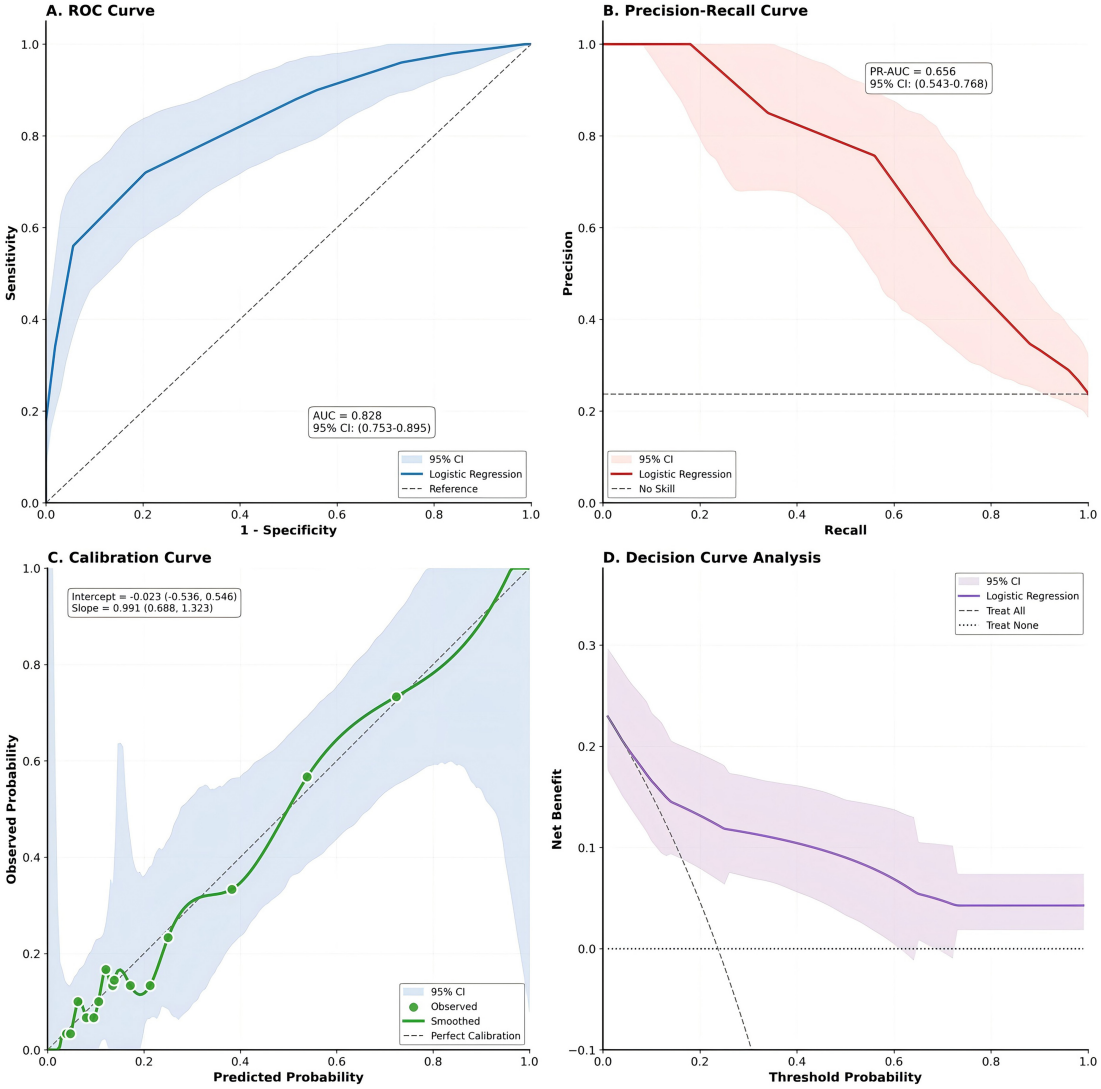
Predictive performance of the final logistic regression model on the independent test set after isotonic regression recalibration. **(A)** Receiver operating characteristic (ROC) curve. **(B)** Precision–recall (PR) curve. **(C)** Smoothed calibration curve. **(D)** Decision curve analysis (DCA). Shaded areas indicate 95% confidence intervals.

The predictive performance of the logistic regression model was thoroughly evaluated on the independent test set using discrimination, calibration, and clinical utility metrics (Table 4 and Figure 4). After isotonic regression recalibration, the model demonstrated moderate-to-good discriminative performance with an ROC-AUC of 0.828 (95% CI: 0.749–0.895) and a PR-AUC of 0.656 (95% CI: 0.533–0.768). Good calibration was observed after recalibration following isotonic regression adjustment, as evidenced by a near-ideal calibration intercept of -0.023 (95% CI: -0.536–0.546) and slope of 0.991 (95% CI: 0.688–1.323), along with a low Brier score of 0.116 (95% CI: 0.087–0.145). Decision Curve Analysis (DCA) suggested potential clinical net benefit across a wide range of threshold probabilities. These results suggest that the recalibrated logistic regression model achieved moderate-to-good internal test-set discrimination and acceptable calibration in this dataset. However, given the learning-curve limitations described above, these performance estimates should be interpreted cautiously and should not be taken as evidence of reliable external generalizability without further validation.

**TABLE 4:** Discrimination and calibration metrics of the LR model on the independent test set.

Metric	ROC AUC	PR AUC	Brier score	Calibration intercept	Calibration slope	ICI	E50	E90
Estimate	0.828	0.656	0.116	-0.023	0.991	0.232	0.138	0.717
CI_lower	0.749	0.533	0.087	-0.536	0.688	0.199	0.138	0.640
CI_upper	0.895	0.769	0.145	0.546	1.323	0.264	0.138	0.750

LR, Logistic Regression; ROC AUC, Area Under the Receiver Operating Characteristic Curve; PR AUC, Area Under the Precision-Recall Curve; ICI, Integrated Calibration Index; E50, 50th percentile of absolute calibration error; E90, 90th percentile of absolute calibration error.

### 3.7 Model interpretation using SHAP

**FIGURE 5:**
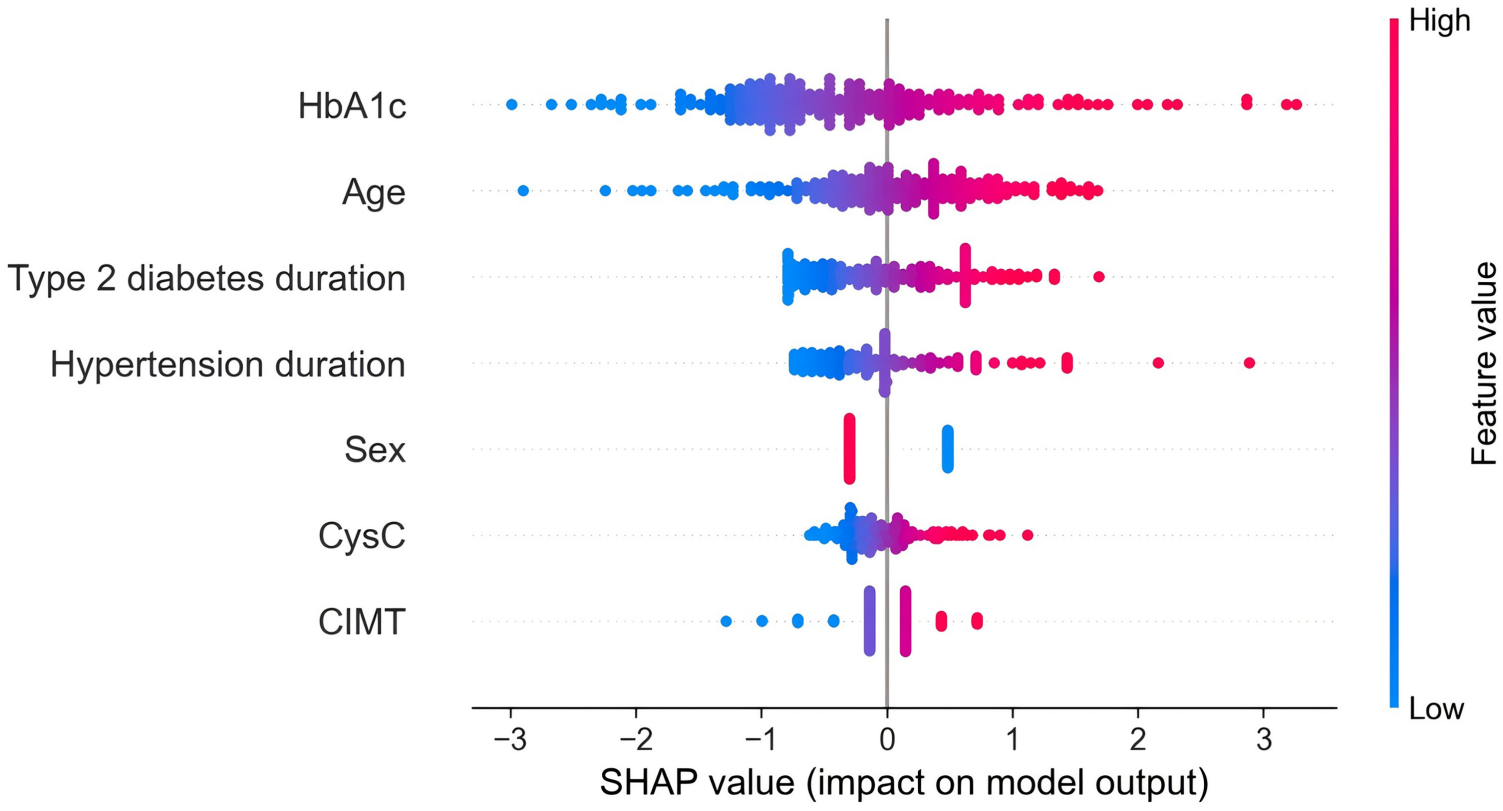
SHAP values for predictors of MACE in LR model.

**FIGURE 6:**
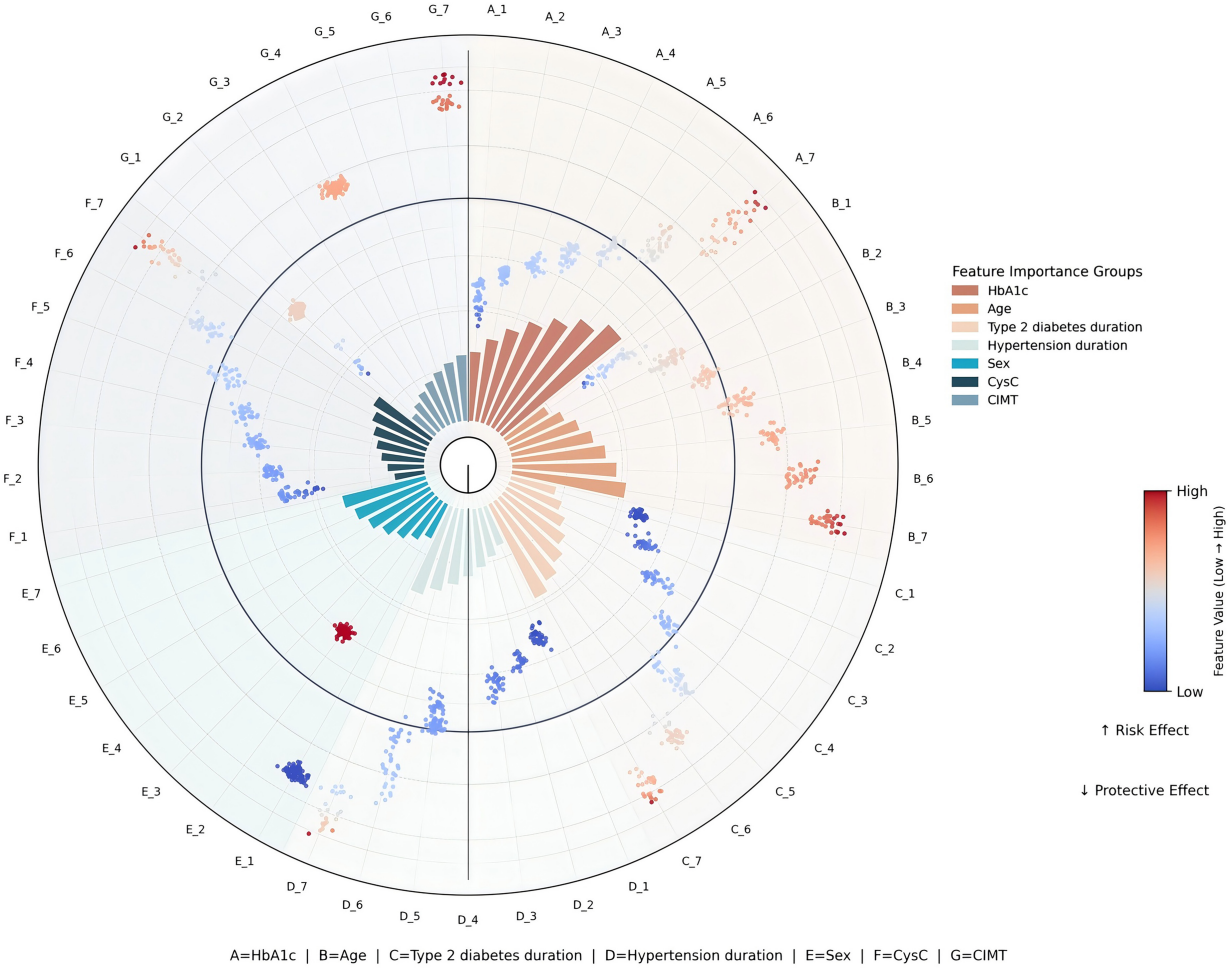
SHAP feature integrated visualization.

SHAP analysis was employed to interpret the contributions of each feature to the logistic regression model’s prediction. The SHAP summary plot (Figure 5) and feature importance ranking revealed that HbA1c, Age, Type 2 diabetes duration, hypertension duration, Sex, CysC, and CIMT were the features in descending order of importance. Specifically, higher values of HbA1c, older Age, longer Type 2 diabetes duration, longer hypertension duration, male sex, higher CysC, and higher CIMT were associated with positive SHAP values, pushing the model’s output towards a higher predicted probability of MACE. The integrated circular visualization (Figure 6) further illustrated feature-attribution patterns of these features, with HbA1c demonstrating the largest SHAP contribution on MACE risk prediction.

## 4 Discussion

In this single-center retrospective cohort study of 1,054 hospitalized patients with T2DM and HTN, we developed and validated an interpretable machine learning model for predicting 1-year MACE. The 1-year MACE incidence was 23.6%, markedly higher than rates reported in community-based studies (28), reflecting the greater baseline disease severity and comorbidity burden in hospitalized patients. Logistic regression was selected as the final model because it showed the best relative balance between discrimination, calibration, and simplicity on the validation set. On the independent test set, the recalibrated model achieved an ROC-AUC of 0.828 and a Brier score of 0.116. However, these results should be interpreted in light of the learning-curve limitations, which suggest rapid saturation of the available predictive signal and potentially optimistic internal performance. SHAP analysis linked model predictions to clinically meaningful domains, including glycemic burden, aging, cumulative disease exposure, renal-related risk, and subclinical atherosclerosis.

### 4.1 Clinical characteristics of the MACE phenotype

Patients who developed MACE were significantly older (69 ± 9 vs. 59 ± 11 years) and had considerably longer durations of diabetes (16 vs. 8 years) and hypertension (10 vs. 7 years), confirming the role of cardiometabolic aging and cumulative disease exposure in adverse outcomes (29–31). The MACE group exhibited elevated systolic blood pressure coupled with reduced diastolic blood pressure—a hemodynamic profile indicative of increased arterial stiffness and reduced vascular compliance (32–34). Metabolically, these patients displayed poorer glycemic control (higher HbA1c and fasting glucose) and more pronounced insulin resistance (higher TyG index) (35). In addition, CIMT was higher in the MACE group, suggesting a greater burden of subclinical atherosclerosis and vascular structural remodeling. This finding is biologically consistent with the cumulative vascular injury caused by long-standing hyperglycemia and hypertension, and supports the role of CIMT as an integrated marker of vascular damage in this high-risk population.

Notably, the MACE group had lower BMI 24.2 vs. 24.8 kg/m^2^ and lower albumin 41.1 vs. 43.9 g/L. The lower BMI and albumin levels observed in the MACE group should be interpreted cautiously. Although this pattern may be partly consistent with the obesity paradox described in patients with established cardiovascular disease or heart failure (36, 37), it may also reflect reverse causation. Patients with greater subclinical cardiovascular burden, chronic inflammation, frailty, or nutritional decline may have experienced involuntary weight loss and hypoalbuminemia before hospitalization. Therefore, these findings should not be interpreted as evidence that higher BMI is protective, but rather as a descriptive phenotype that may reflect poorer baseline health status and greater disease severity. This interpretation is supported by significantly elevated inflammatory markers in the MACE group—WBC, neutrophil count, hs-CRP, SIRI, and SII—consistent with an exacerbated state of chronic low-grade inflammation that, when superimposed on compromised nutritional status, can aggravate endothelial injury and accelerate atherosclerosis progression (38–40).

### 4.2 Cystatin C: beyond renal function

The prominence of CysC as an independent predictor of MACE in our study—with an OR of 1.39—merits in-depth discussion. Cystatin C is a 13.3-kDa cysteine protease inhibitor produced at a constant rate by all nucleated cells, freely filtered by the glomerulus, and almost completely reabsorbed and catabolized by proximal tubular cells. These properties make CysC a sensitive endogenous marker of glomerular filtration that is less influenced by muscle mass, age, and sex than creatinine (41, 42). However, increasing evidence indicates that the prognostic value of CysC in cardiovascular disease extends well beyond its filtration function (43, 44).

At the epidemiological level, large-scale individual data analyses have repeatedly confirmed that elevated CysC is associated with higher cardiovascular risk and adverse outcomes, even in individuals with preserved renal function (45, 46). A meta-analysis involving 114 global cohorts and over 27.5 million participants demonstrated that both lower CysC-based estimated glomerular filtration rate (eGFRcys) and higher urine albumin-to-creatinine ratio were associated with increased risks of coronary heart disease, heart failure, atrial fibrillation, and stroke, even at the earliest stages of kidney injury (47). This association is consistent across the general population, individuals with diabetes, and patients with established cardiovascular disease. For instance, in patients with stable coronary heart disease, higher baseline CysC levels were significantly associated with an elevated long-term risk of coronary death or non-fatal myocardial infarction (48); and in critically ill patients with acute myocardial infarction complicated by cardiogenic shock, the CLIP score, which combines CysC, lactate, interleukin-6, and N-terminal pro-B-type natriuretic peptide, outperformed traditional clinical scores in predicting 30-day mortality (49). Although CysC has been associated with cardiovascular outcomes in hypertensive, diabetic, and general cardiovascular-risk populations, evidence in patients with established coexisting T2DM and HTN remains limited. Our findings extend existing evidence by suggesting that CysC may provide additional prognostic information for 1-year composite MACE risk in this high-risk dual-disease population.

Delving into the underlying mechanisms, CysC may not be viewed merely as a passive surrogate marker of glomerular filtration. While serving as a sensitive indicator of early mild glomerular filtration decline and microvascular injury (41, 50), CysC has in recent years been increasingly recognized to directly participate in the processes of microvascular endothelial injury, inflammation, and fibrosis (51). Animal experiments have revealed that CysC deficiency exacerbates the inflammatory response in a lipopolysaccharide-induced sepsis model through mechanisms closely related to enhanced NLRP3 inflammasome activation, upregulated caspase-11 expression, and impaired autophagy (52). This suggests that CysC plays a key role in modulating inflammatory and immune responses. In the context of atherosclerosis, the balance between CysC and cathepsin S is crucial for vascular wall remodeling; in patients with severe peripheral arterial disease, elevated serum CysC and a decreased cathepsin S/CysC ratio reflect a weakening of vascular protective inhibitory mechanisms (53). Furthermore, a recent study demonstrated that asymmetric dimethylarginine and citrulline in the nitric oxide metabolic pathway are significantly associated with cardiovascular events, independently of glomerular filtration rate (whether measured or estimated) (54), suggesting that the link between renal function measures and cardiovascular outcomes may be partially mediated by shared pathways such as endothelial dysfunction. Therefore, elevated CysC may not only reflect a decline in renal filtration function but also directly or indirectly signify a pathological state of inflammation, oxidative stress, and vascular homeostatic imbalance.

The association between elevated CysC and adverse cardiovascular outcomes may involve multiple intertwined pathways. In terms of plaque stability, insufficient compensation or functional inactivation of CysC under oxidative stress may lead to weakened inhibition of cathepsins, such as cathepsin S and K, potentially resulting in excessive degradation of collagen and elastin, thinning of the fibrous cap, and increased susceptibility to plaque rupture and acute coronary events (55). Regarding inflammatory amplification, CysC has been proposed to interact with the NF-κB pathway and may contribute to the release of IL-6, TNF-α, and MCP-1. These inflammatory cytokines may, in turn, influence CysC secretion through JAK/STAT3-related signaling, potentially forming a reciprocal regulatory loop between inflammation and CysC that could contribute to atherosclerosis progression (56, 57). In fibrosis and calcification, the accumulation of elastin degradation fragments associated with altered CysC-related protease–antiprotease balance may promote osteogenic phenotypic changes in vascular smooth muscle cells through TGF-β/Smad-related signaling, potentially contributing to vascular medial calcification. Similarly, alterations in TGF-β/Smad- and Ang II/AT1-related pathways could plausibly contribute to disturbed myocardial collagen turnover, reactive fibrosis, and diastolic dysfunction (58, 59). Long-term coexisting hyperglycemia and hypertension may together promote glomerular hyperperfusion, hyperfiltration, renal microvascular endothelial injury, sustained inflammation, oxidative stress, and progressive fibrosis, thereby contributing to the loss of renal functional reserve (60, 61). In this context, elevated CysC may serve as an integrative marker of microvascular injury and tissue remodeling rather than necessarily acting as a direct causal mediator. Kidney injury itself may further aggravate cardiovascular burden through sodium and water retention, neurohumoral activation, endothelial dysfunction, and increased arterial stiffness (62, 63), potentially acting together with the pathways described above to contribute to adverse cardiovascular outcomes. These pathways provide plausible biological background for the observed statistical association between CysC and MACE, but they should not be interpreted as mechanistic explanations established by the present study.

### 4.3 Model development strategy and methodological interpretation

Our model development pipeline was designed to compare several candidate algorithms under a structured training–validation–test framework while minimizing information leakage. Among the four evaluated algorithms, logistic regression achieved the best relative balance between discrimination, calibration, and model simplicity on the validation set. More complex nonlinear models, particularly random forest and XGBoost, showed greater training–validation divergence and did not provide a clear performance advantage in this dataset.

However, the selection of logistic regression should not be interpreted as evidence of robust external generalizability. The learning curves indicated limited incremental improvement in validation performance as training sample size increased, suggesting rapid saturation of the predictive signal contained in the available routine clinical variables. Therefore, the final model should be viewed as an internally evaluated exploratory model rather than a clinically deployable decision-support tool.

SHAP analysis was used to improve transparency by describing how the original clinical variables contributed to model predictions. At the global level, HbA1c, age, type 2 diabetes duration, hypertension duration, sex, CysC, and CIMT were the main contributors, suggesting that glycemic burden, cardiometabolic aging, cumulative disease exposure, renal-related risk, and subclinical atherosclerosis may be relevant domains for future prognostic research in this population.

### 4.4 Limitations of learning dynamics and generalizability

The learning curve analysis also revealed important limitations that should temper interpretation of the model’s performance. None of the four candidate algorithms demonstrated entirely red-flag-free learning dynamics. Logistic regression showed only a modest increase in validation accuracy as the training sample size increased, indicating rapid saturation of the predictive signal contained in the available routine clinical variables. Although the small training–validation gap for logistic regression is acceptable and does not indicate substantial overfitting, the limited improvement with additional training data suggests that the current feature set may capture only a finite portion of the risk information needed for 1-year MACE prediction.

The nonlinear models showed different but clinically relevant limitations. Random forest and XGBoost exhibited substantial divergence between training and validation performance, consistent with overfitting. The support vector machine model did not show meaningful performance gains as the training sample size increased. Taken together, these findings suggest that the observed test-set performance (ROC-AUC of 0.828, Brier score of 0.116) is likely optimistic and may not generalize reliably to new patients from different clinical settings or time periods.

Therefore, in its current form, this model should not be deployed as a practical clinical decision-support tool. Substantial external validation in independent populations would be required before any clinical application could be considered. The main contribution of this study should be interpreted as a methodological exploration of machine learning approaches for 1-year MACE prediction in hospitalized patients with coexisting T2DM and HTN, and as hypothesis-generating evidence identifying candidate predictors, particularly CysC. The learning curve evidence does not yet support claims of robust generalization sufficient for routine clinical implementation.

#### 4.4.1 Implications

This study has potential implications for future research and model development. First, the association between CysC and 1-year composite MACE suggests that renal-related biomarkers may warrant further investigation in patients with coexisting T2DM and HTN. Elevated CysC should not be interpreted as a standalone decision threshold, but as a candidate prognostic marker that may reflect broader renal–vascular risk. Second, the use of seven routinely available clinical variables suggests that future externally validated models based on routine data may be feasible. However, given the learning-curve limitations and single-center retrospective design, the present model should not be used to guide discharge follow-up or clinical decision-making until its performance has been confirmed in independent prospective cohorts.

#### 4.4.2 Limitations

Several limitations of this study should be considered. First, its single-center, retrospective design may introduce selection bias, and external validation in a multicenter prospective cohort is essential before clinical implementation. Second, the 1-year follow-up period precludes assessment of longer-term predictive performance. Third, unmeasured confounders—including medication adherence, socioeconomic status, and lifestyle factors—may have influenced the observed associations. Fourth, the composite MACE endpoint included etiologically heterogeneous events. Because cause-specific mortality and complete component-level event data were not reliably available, we could not perform competing-risk analysis or sensitivity analyses for individual MACE components. Thus, the model should be interpreted as predicting overall 1-year composite MACE risk rather than specific fatal or non-fatal cardiovascular outcomes.

## 5 Conclusion

In this single-center retrospective cohort study of hospitalized patients with coexisting T2DM and HTN, we conducted a methodological exploration of machine learning approaches for 1-year composite MACE prediction using routinely available clinical variables. LASSO identified HbA1c, age, hypertension duration, CysC, T2DM duration, and CIMT as stable candidate predictors, with sex additionally incorporated based on clinical relevance. Logistic regression showed the best relative balance between discrimination, calibration, and simplicity among the evaluated algorithms and achieved moderate-to-good internal test-set performance after recalibration. However, learning-curve analysis indicated limited incremental improvement with increasing training sample size, suggesting that the available variables may capture only a finite portion of the relevant risk signal and that internal performance estimates may be optimistic. CysC may provide additional prognostic information beyond its conventional role as a renal filtration marker, but this finding should be interpreted as hypothesis-generating rather than causal or mechanistic. The current model should not be considered ready for clinical decision support; external validation and prospective impact studies are required before any routine clinical use.

## Data Availability

The data analyzed in this study is subject to the following licenses/restrictions: The raw data supporting the conclusions of this article will be made available by the authors without undue reservation. The data will be shared on reasonable request to the corresponding author. Requests to access these datasets should be directed to Zhengyi Zhang, 1939115272@qq.com.
